# An integrated cyberGIS and machine learning framework for fine-scale prediction of Urban Heat Island using satellite remote sensing and urban sensor network data

**DOI:** 10.1007/s44212-022-00002-4

**Published:** 2022-09-09

**Authors:** Fangzheng Lyu, Shaohua Wang, Su Yeon Han, Charlie Catlett, Shaowen Wang

**Affiliations:** 1 cyberGIS Center for Advanced Digital and Spatial Studies, University of Illinois at Urbana-Champaign, Urbana, IL USA; 2 Department of Geography and Geographic Information Science, University of Illinois at Urbana-Champaign, Urbana, IL USA; 3 Key Laboratory of Digital Earth Science, Aerospace Information Research Institute, Chinese Academy of Sciences, Beijing, 100094 China; 4 International Research Center of Big Data for Sustainable Development Goals, Beijing, 100094 China; 5 Computing, Environment, and Life Sciences, Argonne National Laboratory, Chicago, IL USA

**Keywords:** cyberGIS, Machine learning, Remote sensing, Urban Heat Island, Urban sensor network

## Abstract

Due to climate change and rapid urbanization, Urban Heat Island (UHI), featuring significantly higher temperature in metropolitan areas than surrounding areas, has caused negative impacts on urban communities. Temporal granularity is often limited in UHI studies based on satellite remote sensing data that typically has multi-day frequency coverage of a particular urban area. This low temporal frequency has restricted the development of models for predicting UHI. To resolve this limitation, this study has developed a cyber-based geographic information science and systems (cyberGIS) framework encompassing multiple machine learning models for predicting UHI with high-frequency urban sensor network data combined with remote sensing data focused on Chicago, Illinois, from 2018 to 2020. Enabled by rapid advances in urban sensor network technologies and high-performance computing, this framework is designed to predict UHI in Chicago with fine spatiotemporal granularity based on environmental data collected with the Array of Things (AoT) urban sensor network and Landsat-8 remote sensing imagery. Our computational experiments revealed that a random forest regression (RFR) model outperforms other models with the prediction accuracy of 0.45 degree Celsius in 2020 and 0.8 degree Celsius in 2018 and 2019 with mean absolute error as the evaluation metric. Humidity, distance to geographic center, and PM_2.5_ concentration are identified as important factors contributing to the model performance. Furthermore, we estimate UHI in Chicago with 10-min temporal frequency and 1-km spatial resolution on the hottest day in 2018. It is demonstrated that the RFR model can accurately predict UHI at fine spatiotemporal scales with high-frequency urban sensor network data integrated with satellite remote sensing data.

## Introduction

More than 50 percent of the human population lives in cities, and this proportion is projected to reach 60% by the end of 2030 with about 5 billion people living in urban areas (DESA, 2002; Zhou et al., 2011). Similarly, urban land cover will increase by 1.2 million km^2^ by 2030 if the current trend persists (Seto et al., 2012). Rapid urbanization has caused many environmental and sustainability challenges in cities and beyond. Urban Heat Island (UHI) effects, featuring significantly higher temperature in parts of metropolitan areas compared to their surrounding areas, have been affecting people living in cities (Baklanov et al., 2016), yet these effects are mostly studied in macroscope, comparing temperature within a city to those in the surrounding suburban areas (Somers et al., 2013). However, temperature is highly variable within an urban area along a gradient of urban development (Somers et al., 2013), with significant differences from one neighborhood to another as affected by urban form (green space, water, residential vs. dense urban, etc.). Consequently, fine-scale UHI detection is required to study temperature among different locations *within* an urban area. Chicago, in the USA, at 600 km^2^ and nearly 3 M residents, is at the heart of  a 10 M population Metropolitan Statistical Area (MSA). The city has diverse land cover types, from dense urban canyons to residential. Adjacent to Lake Michigan, the second largest of the Great Lakes at 58,030 km^2^, the city manages nearly 9,000 acres (36 km^2^) of green space—the largest municipal park system in the USA. The city anchors Cook County, which manages some 70,000 acres (283 km^2^) of forest preserves and parks. Particularly within the city, the diversity of land cover also reflects significant challenges with under-resourced communities resulting from over a century of social and racial segregation issues (Moore, 2016). Consequently, climate change, and UHI, have disproportionate impact on these communities, underscoring the criticality of achieving fine-scale UHI detection by comparing temperature inside the city.

UHI is not only directly responsible for worsening the adverse health effects from exposure to extreme thermal conditions (Tan, 2010), but also exacerbates air pollution (Li, 2018), adding to the burden on specific communities within cities. Therefore, it is important to understand UHI effects within a city for improving the health and wellbeing of urban population. Researchers in diverse domains have used thermal remote sensing images from satellites to study UHI, which had to resolve the issues of low temporal frequency (Lo et al., 1997; Szymanowski & Kryza, 2009). With the widespread implementation of location-aware and near real-time sensors in large cities such as Chicago, spatiotemporal data from such sensors can accurately reflect the changes of dynamic urban environments (Wang et al., 2021). Supported by remote sensing and high-frequency urban sensor network data, this research aims to address the following two research questions: 1) how to predict UHI within a city at fine spatial and temporal scales? 2) how to integrate high-frequency urban sensor network data and remote sensing data to achieve such prediction using machine learning. This study explores these questions in Chicago using multiple machine learning models (e.g., Artificial Neural Network (ANN), Support Vector Machine (SVM), and Random Forest Regression (RFR)) that are integrated into a cyber-based geographic information science and systems (cyberGIS) framework (Wang, 2010). This framework is developed to predict spatiotemporal distributions of UHI using high-frequency urban sensor network data retrieved from the Array of Things (AoT) (Catlett et al., 2017) and Landsat 8 Collection-2 Level-2 remote sensing satellite imagery focused on Chicago.

As the extensive body of prior UHI studies were conducted using thermal remote sensing data from satellites like Landsat and Aster that record measurements for the same location weekly or bi-weekly, data availability is inadequate to take advantage of machine learning models for fine-scale characterization of UHI. The temporal aspect of UHI was often not adequately addressed due to the data limitation. The cyberGIS framework aims to fill this gap by predicting UHI within Chicago at fine spatial and temporal scales. The framework also is designed to gain better understanding about the relationships between UHI and multiple environmental factors such as air quality indicators (e.g., particulate matter 2.5 (PM_2.5_) concentration), humidity, light intensity, and land surface characteristics.

## Related work

UHI, a phenomenon involving increased air temperature of a city compared to the surrounding area, causes increased energy use and health problems (Oh et al., 2020). Especially in megacities, it is important to understand spatial and temporal patterns of UHI within a city as urban temperature is different across space and over time (Somers et al., 2013). In Chicago—our study area—despite the cooling effects of the Lake Michigan, urban parks, and green spaces, approximately 25 percent of the urban area experienced UHI effects (Alfraihat et al., 2016). Many factors, including population increase and precipitation change (Zhao et al., 2014), unhealthy air quality (Li, 2018), change of thermal properties of building materials in urban areas (Mohajerani et al., 2017; Stempihar et al., 2012), impervious surfaces caused by decrease in urban albedo (Yang et al., 2015), and increase in urban land use transformation (Li, 2021), are possible contributors to UHI effects. Combined with global warming, the expanding urban population, especially those who live in central areas of megacities, not only experience significantly higher summer temperatures but also suffer from adverse health conditions (Tan, 2010) and Urban Pollution Island (UPI) (Li, 2018) side effects of UHI. Machine learning methods including artificial neural network, support vector machine, random forest model, and fuzzy time series have been used to better understand such effects (Oh et al., 2020; Radhika & Shashi, 2009; Chen and Hwang, 2000, Gardes et al., 2020).

**Fig. 1:**
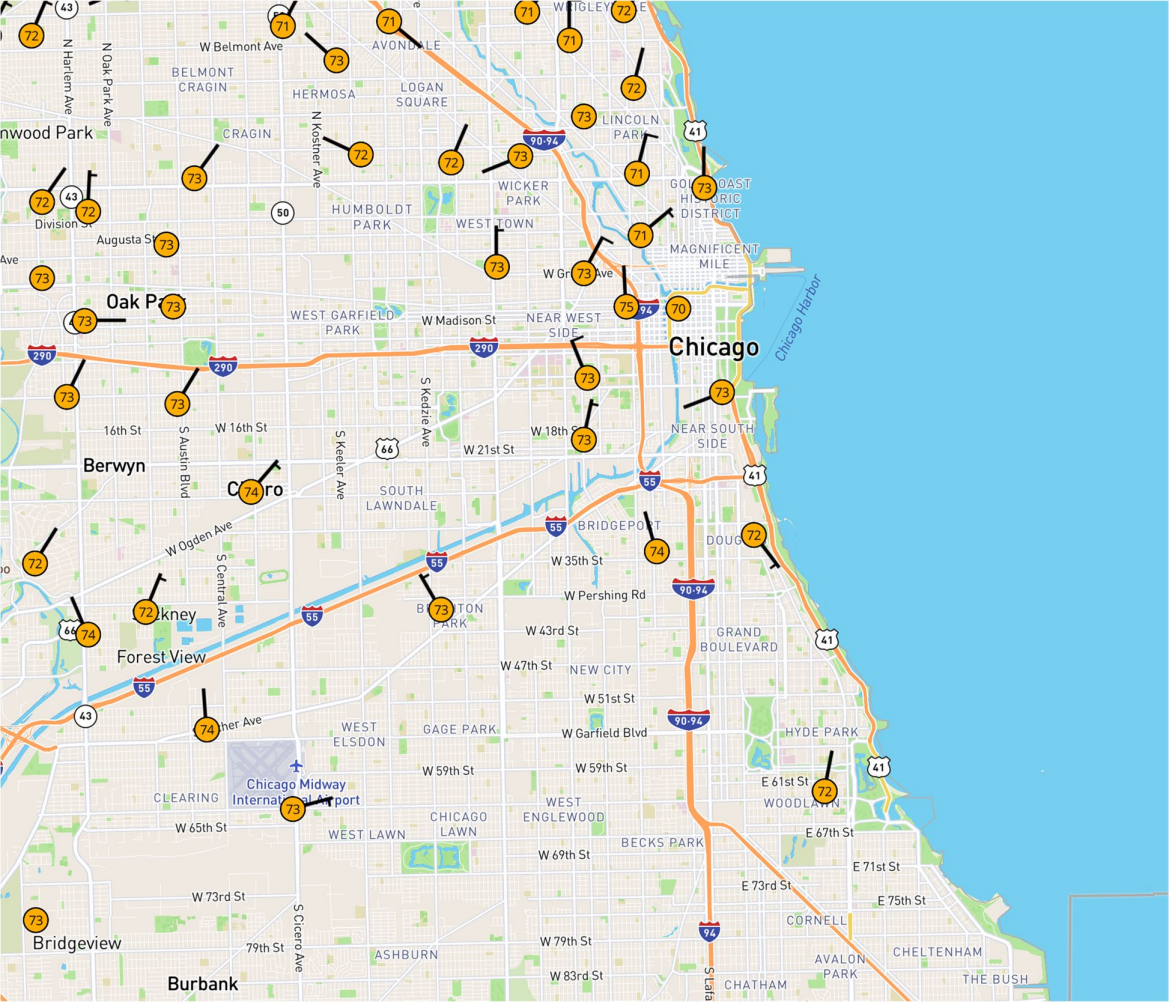
Weather Underground, among the densest weather station networks, is absent in vast regions on Chicago’s South and West sides. (Source: www.wunderground.com)

Spatial and temporal resolutions are critical for predicting UHI in urban areas (Li et al., 2013). Yet temperature is not measured with the spatial or temporal scales necessary to reveal the spatiotemporal dynamics of neighborhood-scale UHI. This is especially true in lower income, higher-minority regions of cities like Chicago. For example, perhaps the densest weather network is Weather Underground, and this shows virtually no weather stations on the South and West sides of Chicago—where over half of the city’s population resides (Fig. 1).

As human health is sensitive to even small temperature changes, there is a demand for fine spatiotemporal granularity prediction of UHI. To achieve fine-resolution spatial delineation of UHI, previous research (Shen et al., 2016; Shi et al., 2018) studies have employed land use regression models, along with multi-temporal and multi-sensor remote sensed data. However, the temporal limitation remains challenging as UHI is modeled using weekly- or biweekly thermal remote sensing imageries. During the past few decades, with the rapid advances in location-aware devices and sensors, urban sensor networks have been deployed to actively collect multi-dimensional data with fine temporal granularity (Armstrong et al., 2019; Li et al., 2021). Urban sensor networks, which have been used to actively monitor air quality, predict crime, record traffic volume (Boyle et al., 2013; Lee et al., 2006; Mead et al., 2013; Nellore & Hancke, 2016; Rathore et al., 2016; Fan et al., 2021) are used in this study to achieve prediction of UHI clusters with fine spatial and temporal granularity.

As urban sensors collect massive high-frequency and multi-dimensional data, harnessing such dynamic data requires novel geospatial data science approaches. Many platforms, including for example PlanetSense, are developed to handle spatial and temporal analysis of such big data (Thakur et al., 2015). In this paper, the data collected from urban sensors (over 500 GB) is handled using cyberGIS-Jupyter (Yin et al., 2019). As a new generation of GIS based on advanced cyberinfrastructure representing a frontier of geospatial data science, cyberGIS comprises a seamless integration of advanced cyberinfrastructure, GIS, and spatial analysis and modeling capabilities while leading to widespread research advances (Anselin & Rey, 2012; Kang et al., 2020; Lyu et al., 2021; Wang, 2010;  Wang, 2016; Wang & Goodchild, 2019). Our cyberGIS framework supports computational reproducibility by integrating our scientific workflow and related data into a cyberGIS-Jupyter notebook that takes advantage of high-performance computing resources (Lyu et al., 2019).

## Data

Chicago is selected as the study area. Although the city of Chicago benefits from Lake Michigan, especially by the lake breeze as a UHI mitigator, the city still suffers from UHI effects (Sharma et al., 2016). Moreover, it is predicted that future heatwaves in Chicago will be more intense, more frequent, and longer lasting in the second half of the twenty-first century (Meehl and Tebalde, 2004). To forecast and analyze UHI effects in Chicago, our study integrates both urban sensor network data and satellite remote sensing data.

**Fig. 2:**
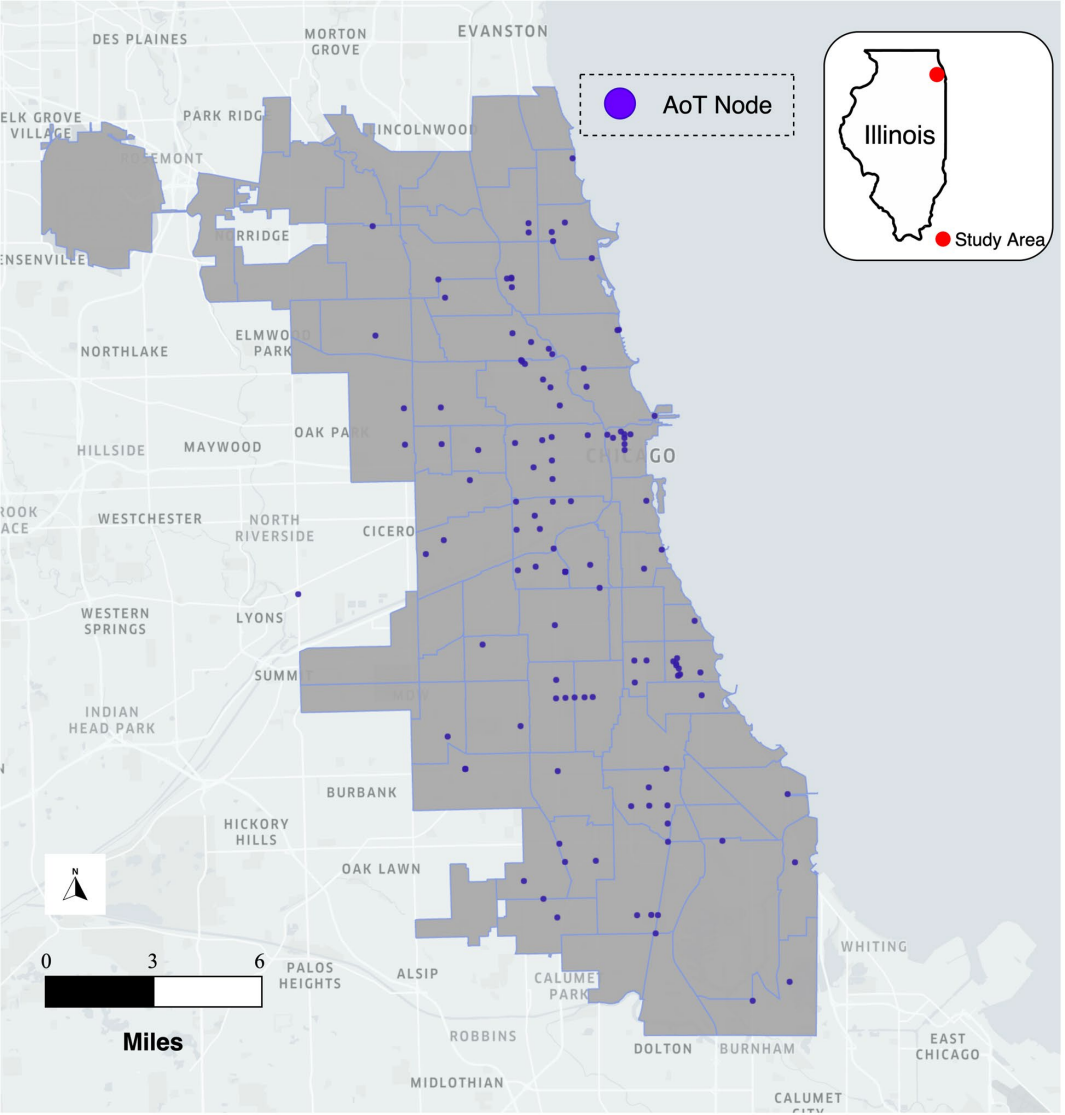
Network of 130 Array of Things (AoT) intelligent sensor nodes in Chicago

With more than 130 nodes deployed in Chicago by the end of 2019, AoT is a sensor network that aimed to collect high-frequency data on urban environments, infrastructure, and activities (Catlett et al., 2017). As shown in Fig. 2, AoT nodes were distributed across the city of Chicago, with each node including both sensors and embedded computing resources to analyze images from sky-facing and ground-facing cameras. From 2016 through 2020, the AoT nodes collected data including temperature, relative humidity, barometric pressure, light, vibration, carbon monoxide, nitrogen dioxide, sulfur dioxide, ozone, and ambient sound pressure with a time interval of about 30 s (Wang et al., 2021, Catlett et al., 2022). From nearly 4.2 billion measurements collected during its 5 years of operation in Chicago, our study focuses on the summer periods (June 21^st^ to September 23^rd^) from 2018 to 2020. Due to an insufficient number of AoT nodes deployed in the first phase of the AoT project, 2016 and 2017 are excluded from this study. Another data source used in this study is satellite remote sensing data. In particular, Landsat 8 Collection-2 Level-2 data covering the city of Chicago during the summer of 2018 to 2020 are used to provide important information regarding the surrounding physical microenvironment of each AoT node. Landsat-8 Collection-2 Level-2 data provides high-quality images that have gone through geometric-related preprocessing including Terrain Precision Correction, Systematic Terrain Correction, and Geometric Systematic Correction as well as atmospheric correction using the Landsat Ecosystem Disturbance Adaptive Processing System (LEDAPS) and Land Surface Reflectance Code (LaSRC) surface reflectance algorithms (USGS, 2020; USGS, n.d.a; USGS, n.d.b). All 7 bands available are used to describe the physical environment of the study area. However, as the temperature is measured with AoT sensors instead of the Landsat Surface Temperatures (LSTs) from Landsat-8 images, the LSTs are not used in this study. Further, we filter out the remote sensing image tiles with cloud cover larger than 10% to make sure the physical environments of the study area are well-described by the remote sensing data. About 12 GB of Landsat 8 Collection-2 Level-2 remote sensing data that were collected biweekly are used in this study.

Among all the data attributes obtained with AoT nodes and satellite remote sensing imagery, Table 1 shows a selected number of attributes used in this study. The dependent variable is temperature that we aim to predict. The independent variables are organized into four categories: 1) environment variables including relative humidity and light intensity measure of the microenvironment around each AoT sensor; 2) air quality variables including PM_2.5_, sulfur dioxide (SO_2_), and 10 μm particles are hypothesized to have a positive correlation with UHI effects; 3) physical environmental variables including Band1 to Band7 values collected from Landsat 8 Collection-2 Level-2 and the Euclidean distance between each AoT node to the geographic center of the city of Chicago (Hagan, 2019); 4) temporal variables including the time of day and day of year recording the timestamps when data measurements were taken. An independent variable is selected if the variable has been proven to have correlation with UHI formation by previous work in literature and there are sufficient reliable data captured at different times. All the attributes listed in Table 1 are used as input to fit and predict temperature and UHI clusters in this study. Different AoT sensor configurations listed in Table 1 can be found at the AoT data download site, https://github.com/waggle-sensor/sensors/tree/master/sensors/datasheets.

**Table 1:** Summary of Variables

Variable	Definition	Unit	Measurement	Source
*Dependent variable: temperature measured by AoT sensors*				
TEMP	Temperature	°C	AoT	AoT sensors: *bmp180, htu21d, pr103j2, tmp112, tsys01, hih6130, htu21d*
*Independent variable: environment variable*				
HUMD	Relative humidity	RH	AoT	AoT sensors: *hih4030, htu21d, hih6130*
Light	Light intensity	uW/cm^2	AoT	AoT sensors: *tsl260rd, mlx75305, tsl250rd*
*Independent variable: air quality variable*				
PM_2.5_	Particles with diameters 2.5 µm and smaller	μg/m^3	AoT	AoT sensors: *opc_n2, psm7003*
SO_2_	Sulfur dioxide concentration	ppm	AoT	AoT sensors: *3SP_SO2_20*
10 μm	Microparticle in diameters less than 10 μm	μg/m^3	AoT	AoT sensors: *Psm7003*
*Independent variable: physical variable*				
B1 – B7	Band value from 1 to 7 with wavelength	W/m2/μm	Pixel (30 m × 30 m)	Landsat 8 Collection-2 Level-2
DoC	Euclidean distance between each AoT node to the geographic center of the city of Chicago	m	Distance	Map
*Independent variable: temporal variables*				
ToD	Time of day	minutes	AoT	AoT timestamp
DoY	Day of year	day	AoT	AoT timestamp

## Method

**Fig. 3:**
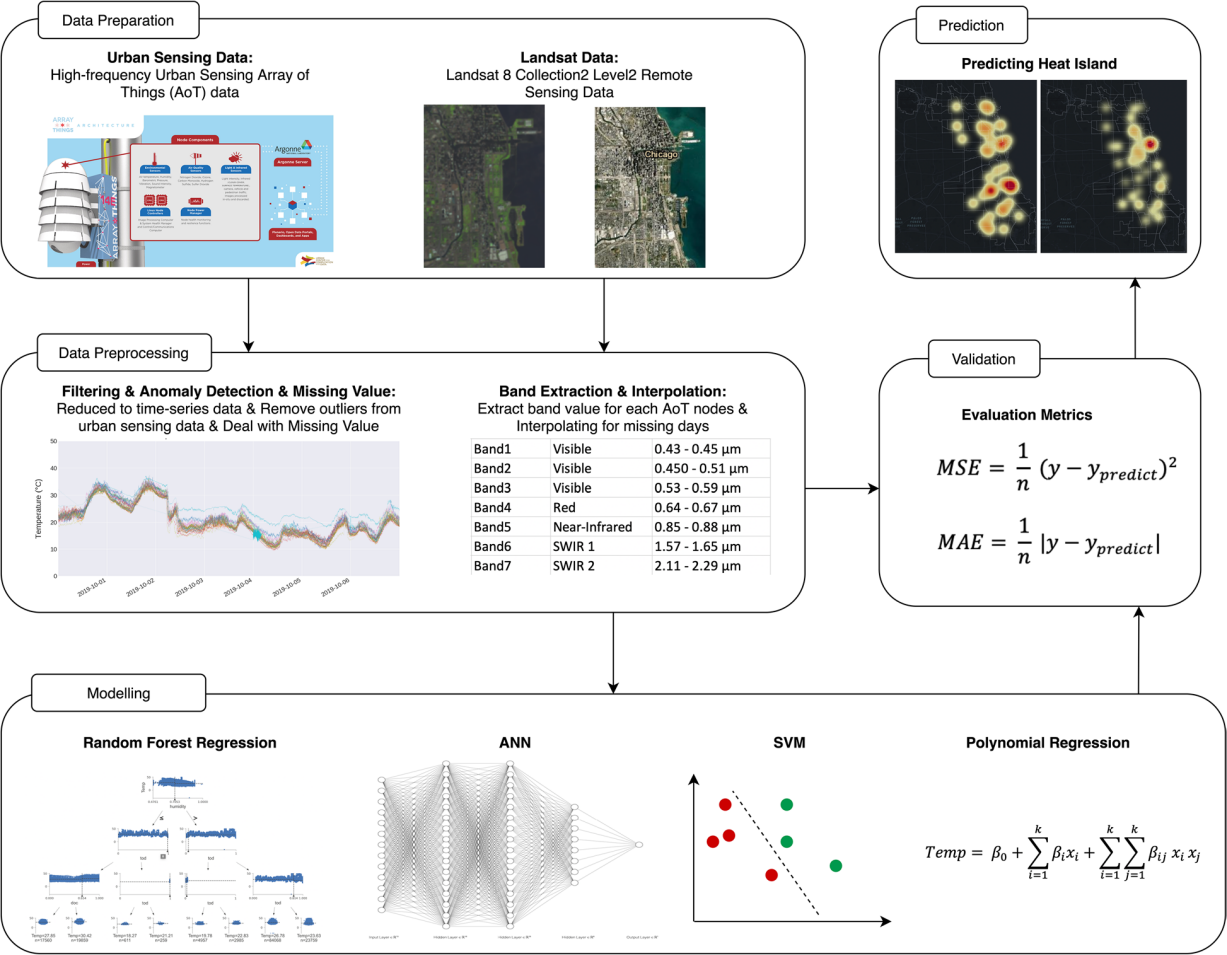
cyberGIS framework

Our method is centered on a cyberGIS framework for integrating multiple machine learning models into a multi-step workflow encompassing five major components – data preparation, data preprocessing, modeling, validation, and prediction. As shown in Fig. 3, the high-frequency urban sensing data is collected from the AoT urban sensor network with a temporal frequency of 26 s on average (Wang et al., 2021). Combined with remote sensing data collected from Landsat 8 (Collection-2 Level-2), we further process the urban sensing data by doing data filtering, anomaly detection, and missing value interpolation. For Landsat data, we extract the band value, which is the Digital Number (DN) of the band, for the location of each AoT node. As the temporal granularity for physical environment indicators measured by remote sensing images are coarse especially compared with the AoT sensor data, a linear interpolation is conducted on the weekly or bi-weekly collected remote sensing data to generate daily remote sensing images and corresponding DN as physical environment indicators. RFR, ANN, SVM, and polynomial regression are used in predicting UHI in Chicago. To evaluate the performance of each model, Mean Square Error (MSE) and Mean Absolute Error (MAE) are used as metrics. In the last step, cartographic maps and 3-D visualization of fine spatiotemporal granularity representation of predicted UHI are integrated into the workflow. Computational reproducibility is supported using a cyberGIS platform where all the code, data, and required software libraries are maintained for reproducing this study.

### Data preprocessing

Data preprocessing was conducted using cyberGIS-Jupyter. First, the AoT data, which exceeds 500 GB in size, is filtered based on their geospatial location and time periods for this study that focuses on the summers of 2018, 2019, and 2020. Second, the high-frequency data is reduced into time-series data that has a time interval of 10 min. The data is first segregated based on its node location before being reduced into time-series data. Each sensor’s attributes are the average of all values under that time span recorded by the same sensor. Then, the anomaly values, those with a temperature that have erroneous records found in the raw AoT data, or beyond the existing boundary of each sensor or the predefined cutoff values are removed. After filtering out those abnormal values, further outlier detection methods are applied to the values from different sensors from the same node at the same time to get the outlier cutoff value. Here, since there is a situation where there are multiple sensors in one node monitoring the same attributes at the same time, there is a need for anomaly detection to filter out the erroneous values. The fence is defined as: *[Q*_*1*_* – 1.5IQR, Q*_*3*_ + *1.5IQR],* where Q_1_ is the first quantile, Q_3_ is the third quantile, and IQR is the difference between Q3 and Q1 (Rousseeuw & Hubert, 2011). After filtering out the outliers, the valid values from different sensors are aggregated as their mean value and the output of the attributes for one certain AoT node at that time. While processing AoT data, another computing thread working with Landsat 8 Collection-2 Level-2 data is executed in parallel. For each AoT node, the band values from the remote sensing image pixel that contains the node are extracted based on the location of each AoT node to represent the physical microenvironment. Since the remote sensing imageries are available bi-weekly in our study, the band values are extracted using linear interpolation with remote sensing imageries from the two closest days available.

The last step for data preprocessing is data integration, where the processed AoT data is merged with the remote sensing imagery data based on their geographic locations. However, the integrated data cannot be used directly as an input to the machine learning models due to the existence of missing values. Especially for the AoT dataset, not all types of sensors are equipped on each AoT node and there was often a time when certain sensors on a node were not functioning. To deal with missing values, a random forest-based Multivariate Imputation by Chained Equations (MICE) method is used to fill in the missing values (Wilson, 2021). MICE is a state-of-the-art method for treating complex incomplete data and is often used for numeric data to resolve missing values (Buuren & Groothuis-Oudshoorn, 2011).

### Model and validation

Due to computational intensity of handling the large dataset, machine learning model training was conducted using Bridges-2 – a high-performance computer at the Pittsburgh Supercomputing Center. Graphics processing unit (GPU) Tesla v100 is equipped within Bridges-2 for model training. After normalizing the independent variables (Table 1), the dataset is randomly divided into 80 percent training data and 20 percent testing data.

Polynomial regression is straightforward as we fit the regression model with the equation below:

$$Temp= {\beta }_{0}+\sum_{i=1}^{15}{{\beta }_{i}x}_{i}+\sum_{i=1}^{15}\sum_{j=1}^{15}{\beta }_{ij} {x}_{i }{x}_{j}+ \varepsilon$$

where Temp is temperature, which is the target function, the total number of independent variables is 15 (Table 1), ${x}_{i}$ is the value corresponding to the ${i}^{th}$ attribute and ε is the residual variable from the model. The polynomial regression model serves as a baseline for the prediction. Compared with machine learning models, polynomial regression is relatively straightforward. Thus, the performance of our chosen machine learning models can be evaluated by comparing them with this polynomial regression model. ANN is designed with 3 hidden layers. As other researchers have used ANN for predicting UHI effects (Oh et al., 2020), ANN can serve as a base line for our model validation. In addition, SVM and RFR are incorporated into the framework of this study. To avoid overfitting, we choose 16 as max depths for the RFR model as we are dealing with 15 independent variables.

To evaluate the performance of each model, Mean Square Error (MSE) and Mean Absolute Error (MAE) are adopted as evaluation metrics:

$$MSE= \frac{1}{n} {(temp-{temp}_{predict})}^{2}$$

$$MAE= \frac{1}{n} |temp-{temp}_{predict}|$$

where $temp$ is the target temperature in the testing sample and ${temp}_{predict}$ is the temperature predicted with our framework.

### Prediction

Fine spatiotemporal granularity prediction of temperature and spatiotemporal clusters of UHI in Chicago is conducted with the best-performing machine learning method. 1 km and 10 min are selected as spatial and temporal resolution respectively. For each spatiotemporal point, the urban sensor-related independent variables are estimated using inverse-distance weighing (IDW) spatial interpolation based on the values of nearby AoT nodes. Remote sensing imagery-related independent variables are estimated daily using linear interpolation based on the two most recent remote sensing imageries covering Chicago at the location we are interested in.

## Result

First in Sect. 5.1, the validation of each machine learning model is conducted to identify the best machine learning model. Then in Sect. 5.2, fine spatiotemporal granularity prediction of UHI in Chicago is described.

### Validation

The testing metrics of polynomial regression and machine learning models are shown in Table 2. The RFR model outperforms the other machine learning models and the regression model in all three years based on both MAE and MSE. The MAE of RFR in all three years ranges from 0.45 to 0.8 degrees Celsius while MSE ranges from 0.4 to 1.3 square degrees Celsius. First, the evaluation results of the RFR model are positive. Even in 2018, when the RFR model performs the worst, the evaluation result is still acceptable. The MAE is less than 0.8, showing the average difference between predicted temperature and the actual temperature monitored by urban sensors is less than 0.8. Given the fact that the mechanism underlying the formation of UHI remains unclear and complicated, prediction accuracy with MAE less than 0.8 and MSE less than 1.3 is better compared with the benchmark from Amato et al. (2020) where the MAE is 1.15 degree Celsius. Second, the evaluation result in 2020 is slightly better than the results in 2018 and 2019, which could be caused by the reduction of human activities during the COVID-19 pandemic. In this study, we consider the environmental, physical, temporal aspects as well as variables related to air quality to predict temperature in the microenvironment. One factor we did not take into explicit consideration is human activities due to the limitation of high-frequency human activities data. It is understood that there is a positive correlation between UHI effects and human activities (Lai & Cheng, 2010; Xie et al., 2010). However, during the pandemic in the US, there was a travel restriction on individuals and consequently human activities decreased in the summer of 2020 compared with 2018 and 2019. There is evidence that such lockdowns and travel restrictions triggered by COVID-19 pandemic had a significant impact on the heat emission and air quality indicators, which are used as input in this study (Wong et al., 2021). That might be a reason why we got better evaluation results in 2020 compared with 2018 and 2019 as human activities are not taken into consideration in our model. Last, the RFR model performs consistently well. Admittedly, there is a difference between the evaluation results in three years. However, the difference is not significant, compared with other models like the regression model where the gap of MSE between 2018 and 2020 is about 6.5, the performance of RFR is consistent in all three years.

**Table 2:** Model Evaluation

	**2018**		**2019**		**2020**	
	**MSE**	**MAE**	**MSE**	**MAE**	**MSE**	**MAE**
**Polynomial** **Regression**	13.027	2.837	12.110	2.707	6.645	2.033
**ANN**	12.716	2.747	11.232	2.308	22.201	2.044
**SVM**	11.543	2.649	9.259	2.336	7.752	2.189
**Random Forest Regression**	1.268	0.794	1.24	0.784	0.443	0.455

For 2018 and 2019, both ANN and SVM models outperform the polynomial regression model. However, in 2020, the result with the polynomial regression model is better than the results from ANN and SVM. As the most straightforward model, the polynomial regression model did not perform well in predicting UHI as expected. The R-square value for polynomial regression in 2018, 2019 and 2020 are 0.335, 0.385 and 0.697 and the adjusted *r*-square from 2018 to 2020 are 0.335, 0.384 and 0.696 respectively. Both the *r*-square and the evaluating metrics, including MSE and MAE, show that polynomial regression cannot be used effectively in predicting UHI with the current data we have.

To compare between two machine learning models ANN and SVM, the MSE evaluation result from ANN is generally more significant, especially in 2020. High MSE and relatively low MAE indicate some extreme values predicted by ANN, showing the model can be unstable in the prediction of temperature. Even though the SVM model outperforms ANN with MSE used as the evaluation metric, the ANN outperforms SVM in 2019 and 2020 with MAE as the evaluation metric. The effectiveness of ANN and SVM are considered similar in predicting temperature with the existing dataset. However, these two models are not appropriate to be used in real-world scenarios because their prediction results are mediocre and the RFR model outperforms both models by a large margin. Because human activities could play a significant role in the generation of UHI effects (Lai & Cheng, 2010), the better prediction performance for 2020 than 2018 and 2019 indicated by MAE can be explained by the relative absence of human activities in 2020 due to the COVID-19 pandemic.

**Fig. 4:**
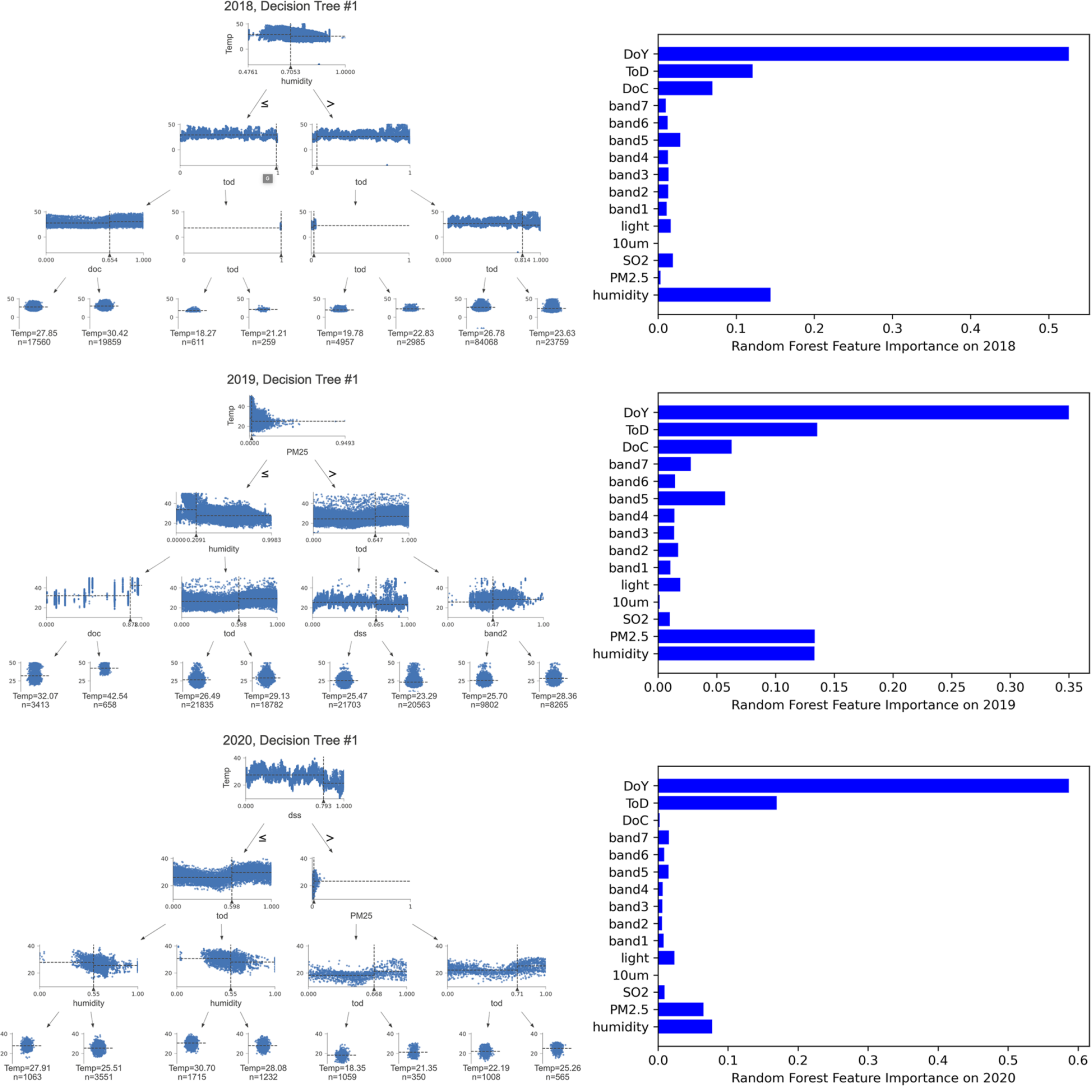
The first three layers of the first decision tree and feature importance in the random forest approach (2018, 2019 and 2020)

As the RFR model performs the best in the validation phase, we investigated the decision trees from the model to understand the model’s functional mechanism. Figure 4 shows the first three layers of the first decision tree and importance of each feature after fitting the RFR model from 2018 to 2020. Figure 4 shows the first 3 layers for the first decision and depicts the contribution of each attribute to the performance of the RFR model. On the three layers of the tree for each fitted model from 2018 to 2020, the attributes of humidity, time of the day, distance to the geographic center, PM_2.5_, day of the year, and band2 from remote sensing imagery play significant roles. Temporal factors, intuitively, are critical in predicting temperature. Other than that, the PM_2.5_ indicator is significant in 2019. Since the humidity attribute is prominent in all three years, it could be a deciding factor for predicting temperature. From the perspective of physical environment variables, the distance to the geographic center variable plays a significant role in the prediction for 2018 and 2019, which can be explained by the higher temperature around the geographic center of the city where the central business district is located in the city of Chicago. Lastly, the band2 attributes are worth noting in the decision tree in 2019. In Landsat 8, Band 2 is the band with a wavelength between 450 to 510 nm. As Band 2 is often used in studies related to vegetation (Acharya & Yang, 2015), vegetation index and greenness of microenvironments could be a potential key to reduction of UHI effects (Imhoff, 2010). Gini importance, which is also known as the impurity-based feature importance, is the total decrease in node impurity averaged over all trees of the ensemble and it is one of the most used method for investigating the importance of features for random forest-based models (Menze et al., 2009). The three most import features, as shown in Fig. 4, are the temporal variables including day of the year and time of the day as well as humidity. In the cases of 2018 and 2019, the distance to the geographic center attribute plays a significant role as well. On the other hand, in 2019 and 2020, PM_2.5_ concentration contributes to the model performance to a relatively large degree.

**Fig. 5:**
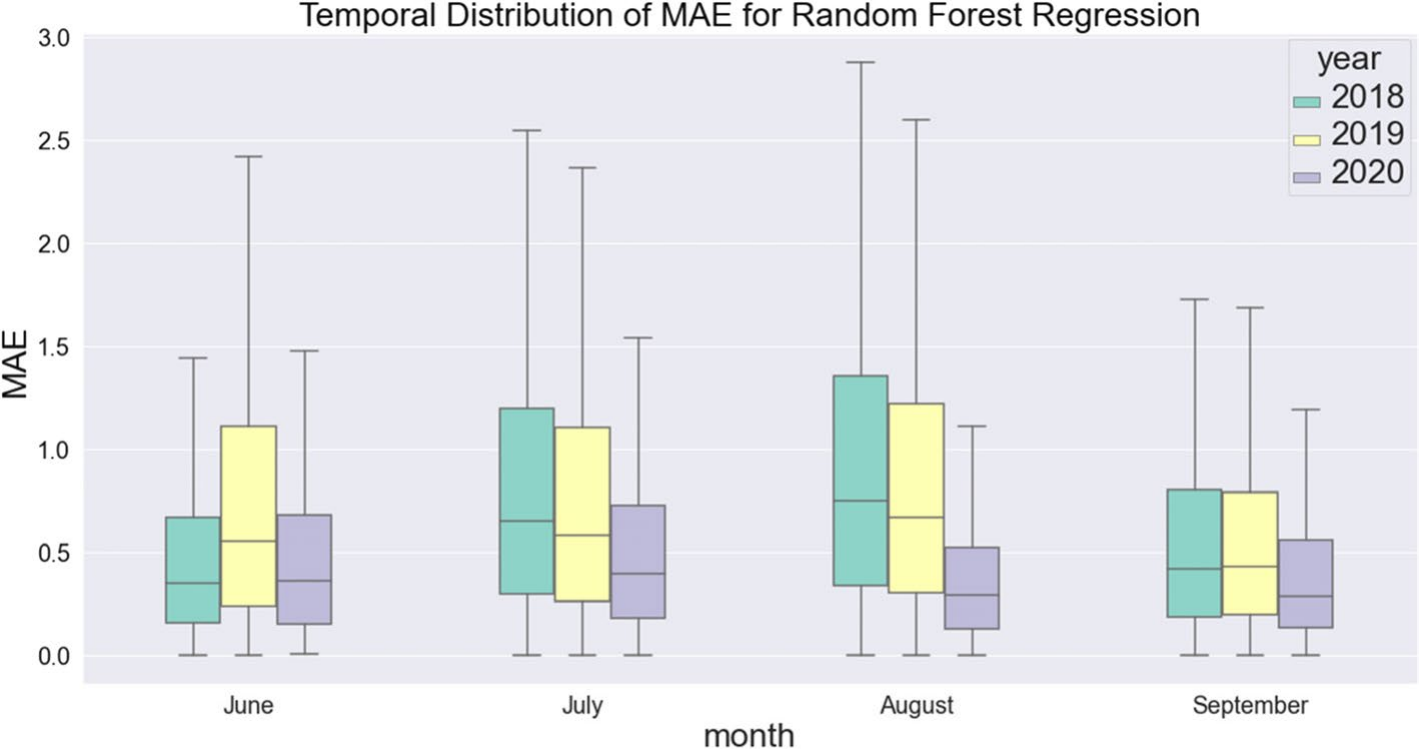
Temporal distribution of MAE for random forest regression

To further evaluate the performance of the RFR model, we analyze the stability of the model in four months in each summer of the selected years from June to September. Figure 5 shows the boxplot of the fitted MAE for the model regarding the four months in each selected year. Even though the boxplot differs regarding different years and different months, the performance of the model is relatively stable, with the median being around 0.5 degree Celsius. Some outliers are likely caused by the noises of the urban sensor network data. Compared with the other methods, including ANN and SVM, which are used in previous studies to predict UHI, the RFR model steadily outperforms in different years and months. While the actual MAE from the RFR model is about 0.8 in 2018 and 2019 and 0.45 in 2020, the performance of the RFR model is stable throughout the summer in each year.

**Fig. 6:**
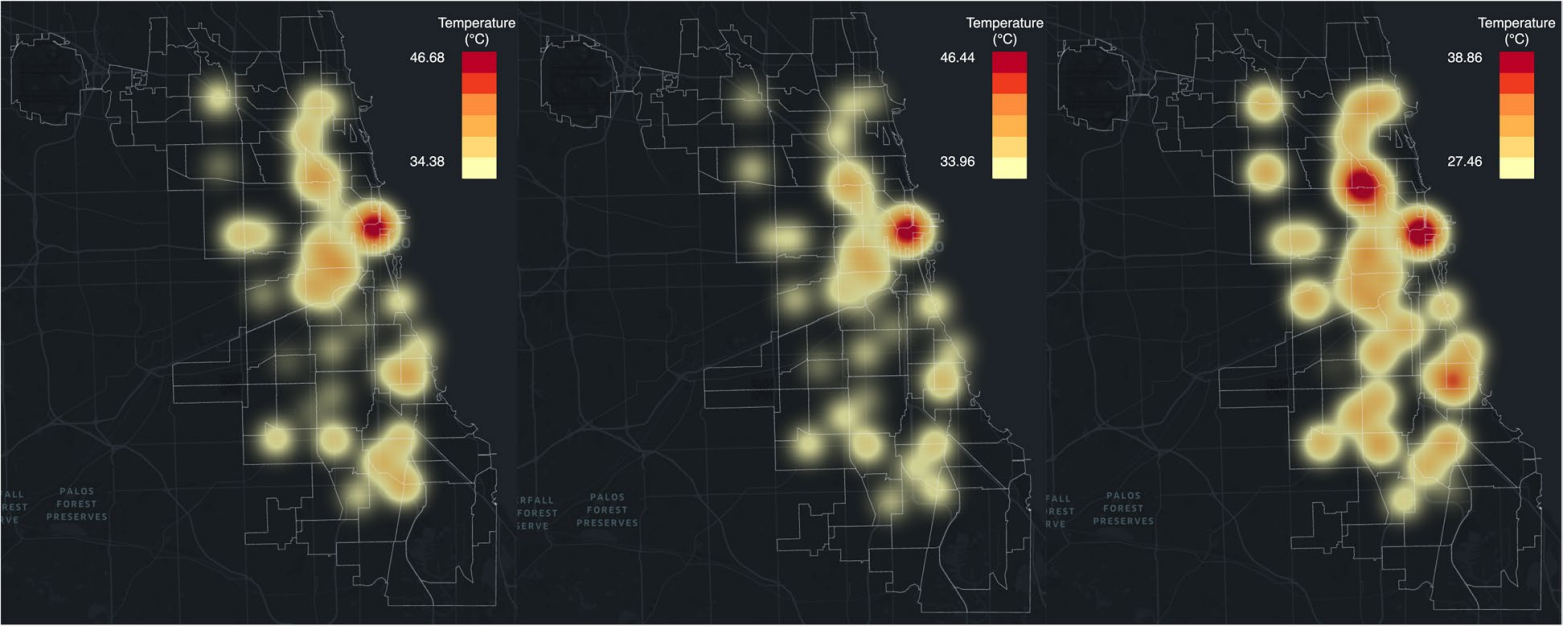
From left to right for 2018.8.27: observed UHI pattern, UHI pattern predicted with RFR, and UHI pattern predicted with ANN

**Fig. 7:**
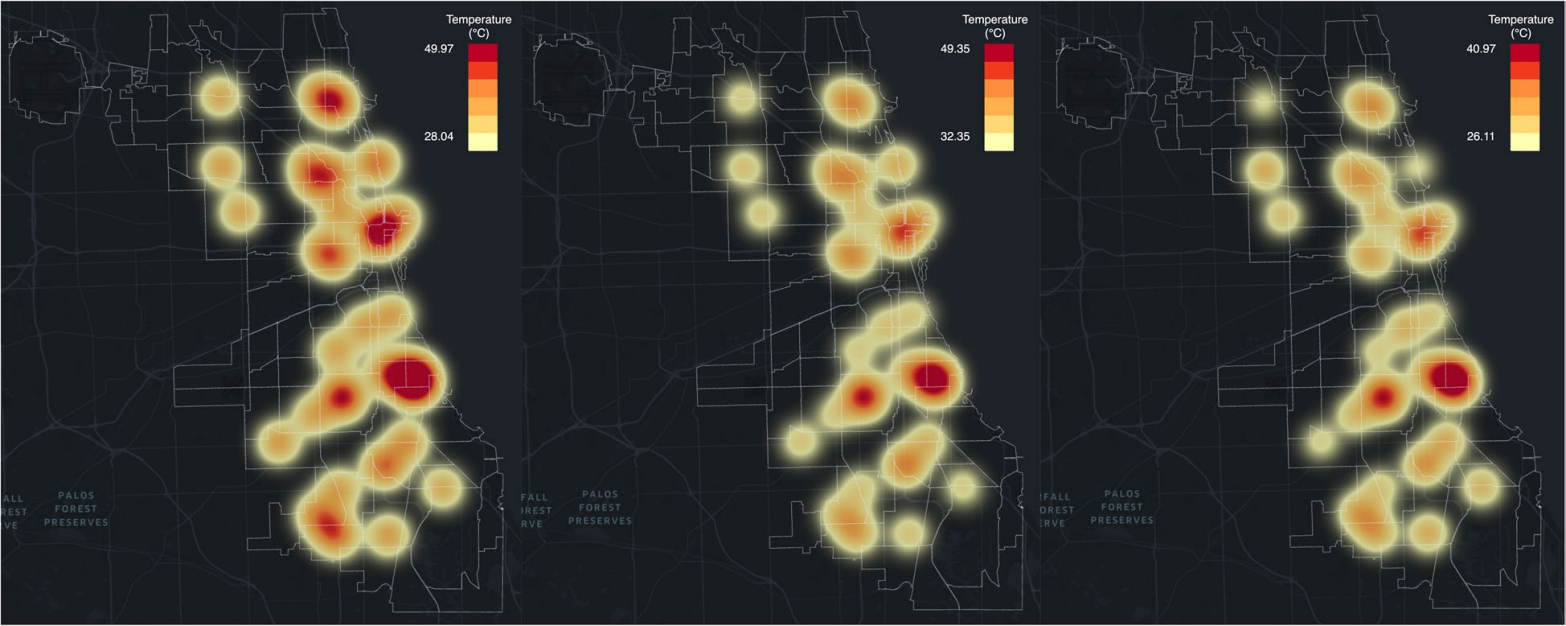
From left to right on 2019.7.20: observed UHI pattern, UHI pattern predicted with RFR, and UHI pattern predicted with ANN

To demonstrate how the RFR model predicts spatial patterns of UHI, we create heatmaps to visualize UHI on the hottest day in Chicago in 2018 and 2019 as shown in Figs. 6 and 7. The hottest day in Chicago in 2018 is August 27^th^, with temperature ranging from 96 degrees Fahrenheit (35.6 degrees Celsius) to 78 degrees Fahrenheit (25.6 degrees Celsius) based on the weather report from the National Oceanic and Atmospheric Administration (NOAA) and AccuWeather. On that day, there were in total 46 functioning AoT nodes available in Chicago.

For all the active AoT nodes in Chicago, based on the highest temperature recorded by each node on August 27^th^, 2018, a heatmap of UHI is generated using the average observed temperature from those AoT nodes based on bilinear interpolation. Similarly, the UHI distribution heatmaps are generated with the temperature predicted with the RFR model and ANN model respectively. Figure 6 shows that the observed heatmap for UHI and the predicted heatmap with RFR is highly consistent. Based on the heatmap generated from the temperature recorded with AoT on the left of Fig. 6, there is a heat island near the loop area of Chicago. The area close to the loop generally has a higher temperature than the surrounding areas. However, the predicted heatmap from the ANN model, which works as a benchmark, is different from the observed heatmap as there are three predicted heat islands located in the loop area of Chicago, the northern part of Chicago, and the southeast part of Chicago.

The same process is applied to the hottest day in 2019 to generate heatmaps for comparison. Based on the weather report from NOAA and AccuWeather, the hottest day in Chicago in 2019 is July 20^th^ with the highest temperature of 96 degrees Fahrenheit (35.6 degrees Celsius) and the lowest temperature on that day being 76 degrees Fahrenheit (24.4 degrees Celsius). On July 20^th^, 2019, there were 39 functioning AoT nodes recording the surrounding environmental attributes including temperature, humidity, PM_2.5_, etc. As we can see from Fig. 7, there are 7 heat islands with the observed data from AoT. Though the predicted heatmap with the RFR model shows a similar pattern, some of the heat islands including two heat islands in the northern part of Chicago and one heat island in the southwestern part of Chicago are not as strong as they are on the observed heatmap. The predicted heatmaps from random forest regression and ANN are similar.

**Fig. 8:**
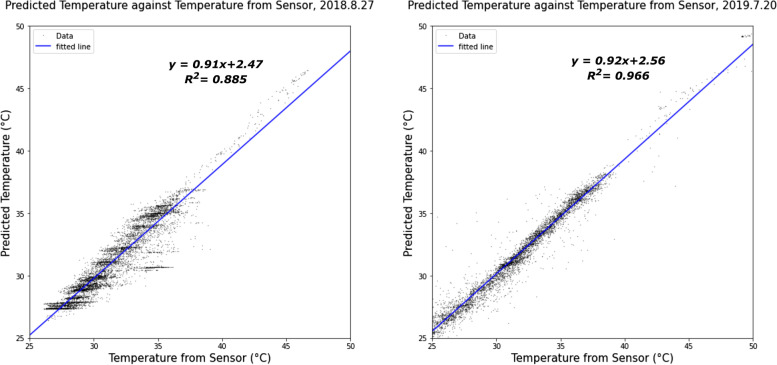
Predicted temperature with the RFR model against temperature detected by AoT sensors on the hottest day in 2018 and 2019 respectively

Figure 8 shows the predicted temperature using the RFR model against the temperature detected by AoT sensors on 2018.8.27 and 2019.7.20. Based on the testing results, we argue that the RRF model can be used to accurately predict temperature with integrated high-frequency urban sensor network and satellite remote sensing data. The RFR model outperforms the polynomial regression model, SVM, and ANN in our case study focused on the city of Chicago.

### Spatiotemporal clusters of UHI

**Fig. 9:**
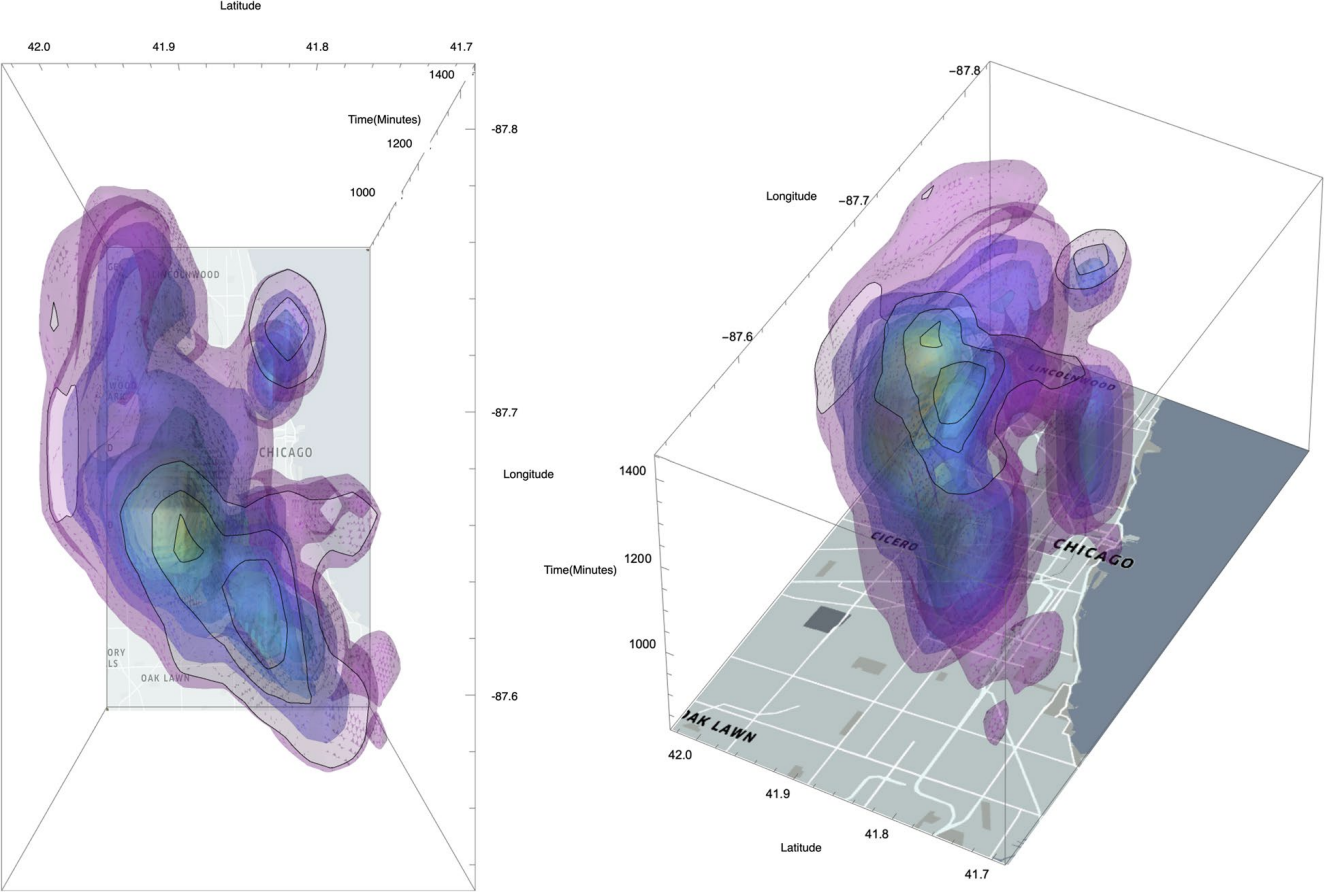
Prediction of UHI clusters in Chicago on 2018.8.27

We apply the RFR model with 1 km as spatial resolution and 10 min as temporal granularity to delineate spatiotemporal clusters of UHI within Chicago on the hottest day in 2018. Spatiotemporal points with extreme high temperature are clustered for visual interpretation. As shown in Fig. 9, the visualization depicts multiple spatiotemporal UHI clusters with fine spatiotemporal granularity. One major spatiotemporal cluster of UHI centered around East Village, Chicago where the latitude is 41.9 and longitude is -87.67 around 3 p.m. in the afternoon. Apart from the major cluster, a minor heat island is detected in the north part of Chicago near Evanston. From the temporal perspective, a UHI cluster was first spotted around 9 a.m. in the downtown area of Chicago and ended around 8 p.m. Around 3 p.m., the temperature reached the highest. Instead of using the temperature recorded in different subareas of the city based on weather reports, our cyberGIS framework provides a way to detect UHI at fine spatiotemporal scales. Especially from the temporal perspective, the framework employed high-frequency urban sensor network data to study the temporal dimension of UHI, which has not been well addressed by previous work.

## Conclusions and future work

This study has developed a framework to integrate cyberGIS and machine learning for fine spatiotemporal granularity prediction of UHI with satellite remote sensing data and high-frequency urban sensor network data. This framework is designed to assess the performance of the polynomial regression model, SVM, ANN, and RFR model in predicting spatial and temporal patterns of UHI in Chicago for the years of 2018, 2019, and 2020. First, the RFR model is found to achieve the best performance among all the machine learning models with MAE being 0.45 degrees Celsius in 2020 and around 0.8 in 2018 and 2019. Humidity, distance to geographic center and PM_2.5_ concentration are found to be important factors contributing to the model performance of RFR model. Second, the RFR model is stable as the performance of the model is consistent during all four months in the summers of 2018, 2019, and 2020. We constructed heatmaps to compare the observed UHI and predicted UHI on the hottest day in 2018 and 2019. The heatmaps show that the predicted spatial patterns are similar to the corresponding patterns from the observed UHI based on the urban sensor network data. Last, the framework is applied to delineate fine-scale spatiotemporal patterns of UHI with 1-km spatial resolution and 10-min temporal resolution using the RFR model on the hottest day in 2018. Our framework has demonstrated that the RFR model can be used effectively to predict spatiotemporal distributions of UHI.

We plan to conduct future work in three aspects. First, human activities are not fully addressed in our study, especially for the travel activities involving vehicles, as they emit not only heat but also exhaust gas, which is believed to cause UHI. Also, manufacturing activities and even the use of electric appliance such air conditioners by city residents may result in temperature increases in some places. As different human activities may contribute to the formation of UHI, the framework could be improved by integrating human activities data. Second, as machine learning models perform better with more high-quality data, the framework could be improved with more sensors and nodes deployed in urban environments. Finally, two AoT follow-on projects are under way that are providing new, near real-time urban sensing data. First, nodes with more powerful edge processors that can be customized with project-specific sensor packages are being deployed to replace AoT nodes in Chicago as part of a National Science Foundation Mid-Scale Research Infrastructure development project called SAGE (Beckman et al., 2019). Second, the AoT team partnered with Microsoft Research, JCDecaux, and the Environmental Law and Policy Center in 2021 to deploy 115 sensor nodes on bus shelters throughout Chicago, each measuring PM_2.5_, temperature, relative humidity, and multiple air pollutant gases (Daepp et al., 2022). Using these and other new data sources, the framework will be enhanced to pursue near real-time prediction of UHI, which is critical to help people living urban areas to be better prepared for extreme heat situations.

## Data Availability

The data, code, and Jupyter notebook for this study are available at https://github.com/cybergis/Urban_Heat_Island. Please read the readme file of the Github repository for retrieving urban sensor network data and remote sensing data used in this study. Another option is to contact the corresponding author for requesting data access.

## References

[CR1] Acharya, T., & Yang, I. (2015). Exploring Landsat 8. *International Journal of IT Engineering and Applied Sciences Research (IJIEASR),**4*(4), 4–10. April 2015.

[CR2] Alfraihat, R., Mulugeta, G., & Gala, T. S. (2016). Ecological evaluation of urban heat island in Chicago City, USA. *Journal of Atmospheric Pollution,**4*(1), 23–29.

[CR3] Amato, F., Guignard, F., Robert, S., et al. (2020). A novel framework for spatio-temporal prediction of environmental data using deep learning. *Science and Reports,**10*, 22243. 10.1038/s41598-020-79148-7 10.1038/s41598-020-79148-7 PMC7746728 33335159

[CR4] Anselin, L., & Rey, S. J. (2012). Spatial econometrics in an age of cyberGIScience. *International Journal of Geographical Information Science,**26*(12), 2211–2226.

[CR5] Armstrong, M. P., Wang, S., & Zhang, Z. (2019). The Internet of Things and fast data streams: Prospects for geospatial data science in emerging information ecosystems. *Cartography and Geographic Information Science,**46*(1), 39–56.

[CR6] Baklanov, A., Molina, L., & Gauss, M. (2016). Megacities, air quality and climate. *Atmospheric Environment,**126*, 235–249.

[CR7] Beckman P, Catlett C, Altintas I, Kelly E, Collis S. (2019). *Mid-scale RI-1: SAGE: A Software-Defined Sensor Network* (NSF OAC 1935984), https://sagecontinuum.org/.

[CR8] Boyle, D. E., Yates, D. C., & Yeatman, E. M. (2013). Urban Sensor Data Streams: London 2013. *IEEE Internet Computing,**17*(6), 12–20. 10.1109/MIC.2013.85 Nov.-Dec. 2013.

[CR9] Buuren, S., & Groothuis-Oudshoorn, K. (2011). MICE: Multivariate Imputation by Chained Equations in R. *Journal of Statistical Software,**45*(3), 1–67.

[CR10] Catlett, C., Beckman, P., Ferrier, N., Papka, M.E., Sankaran, R., Solin, J., Taylor, V., Pancoast, D. and Reed, D., 2022. Hands-On Computer Science: The Array of Things Experimental Urban Instrument. *Computing in Science & Engineering*, *24*(1), 57–63. 10.1109/MCSE.2021.3139405.

[CR11] Catlett, CE., Beckman, PH., Sankaran, R., and Galvin, KK. (2017). Array of things: a scientific research instrument in the public way: platform design and early lessons learned. In: *Proceedings of the 2nd international workshop on science of smart city operations and platforms engineering*. Association for Computing Machinery, New York, NY, USA, 26–33. 10.1145/3063386.3063771.

[CR12] Chen, S., & Hwang, J. (2020). Temperature prediction using fuzzy time series. *IEEE Transactions on Systems Man and Cybernetics Part B (Cybernetics),**30*(2), 263–275. 10.1109/3477.836375 10.1109/3477.836375 18244753

[CR13] Daepp, M., et al. (2022). Eclipse: An end-to-end platform for low-cost, hyperlocal environmental sensing in cities. *2022 21st ACM/IEEE International Conference on Information Processing in Sensor Networks (IPSN). *10.1109/IPSN54338.2022.00010*.*

[CR14] Desa, U. (2002). *World urbanization prospects: The 2001 revision, data tables and highlights*. New York: United Nations Population Division-Department of Economic and Social Affairs, United Nations Secretariat. (ESA/P/WP. 173),.

[CR15] Fan, Y., Zhan, Q., Tang, L., Liu, H., & Gao, S. (2021). Temporal characterization of minute-level PM_25_ variation within a local monitoring network using DWT-DTW. *Building and Environment,**205*, 108221. 10.1016/j.buildenv.2021.108221 ISSN 0360-1323.

[CR16] Gardes, T., et al. (2020). Statistical prediction of the nocturnal urban heat island intensity based on urban morphology and geographical factors - An investigation based on numerical model results for a large ensemble of French cities. *Science of The Total Environment,**737*, 139253. 10.1016/j.scitotenv.2020.139253 ISSN 0048-9697. 32783817 10.1016/j.scitotenv.2020.139253

[CR17] Hagan, C. (2019). *The Heart of The City: Finding Chicago’s Geographic Center*. WBEZ Chicago. https://www.npr.org/local/309/2019/07/15/741161117/the-heart-of-the-city-finding-chicago-s-geographic-center.

[CR18] Imhoff, M. (2010). Remote sensing of the urban heat island effect across biomes in the continental USA. *Remote Sensing of Environment,**114*(3), 504–513.

[CR19] Kang, J. Y., Michels, A., Lyu, F., et al. (2020). Rapidly measuring spatial accessibility of COVID-19 healthcare resources: A case study of Illinois, USA. *International Journal of Health Geographics,**19*, 36. 10.1186/s12942-020-00229-x 32928236 10.1186/s12942-020-00229-x PMC7487451

[CR20] Lai, L. W., & Cheng, W. L. (2010). Air temperature change due to human activities in Taiwan for the past century. *International Journal of Climatology,**30*(3), 432–444. 10.1002/joc.1898

[CR21] Lee, U., Zhou, B., Gerla, M., Magistretti, E., Bellavista, P. and Corradi, A. (2006). Mobeyes: smart mobs for urban monitoring with a vehicular sensor network. *IEEE Wireless Communications*, 13(5), 52–57. 10.1109/WC-M.2006.250358.

[CR22] Li, H. (2018). Interaction between urban heat island and urban pollution island during summer in Berlin. *Science of The Total Environment,**636*, 818–828. 29727848 10.1016/j.scitotenv.2018.04.254

[CR23] Li, M., Liu, J., Lin, Y., Xiao, L., & Zhou, J. (2021). Revitalizing historic districts: Identifying built environment predictors for street vibrancy based on urban sensor data. *Cities,**117*, 103305. 10.1016/j.cities.2021.103305 ISSN 0264-2751.

[CR24] Li, X., Zhou, W., & Ouyang, Z. (2013). Relationship between land surface temperature and spatial pattern of greenspace: What are the effects of spatial resolution? *Landscape and Urban Planning,**114*(2013), 1–8.

[CR25] Lo, C. P., Quattrochi, D. A., & Luvall, J. C. (1997). Application of high-resolution thermal infrared remote sensing and GIS to assess the urban heat island effect. *International Journal of Remote Sensing,**18*(2), 287–304. 10.1080/014311697219079

[CR26] Lyu, F., Xu, Z., Ma, X., Wang, S., Li, Z., & Wang, S. (2021). A vector-based method for drainage network analysis based on LiDAR data. *Computers & Geosciences,**156*, 104892. ISSN 0098-3004.

[CR27] Lyu, F., Yin, D., et al. (2019). Reproducible hydrological modeling with cyberGIS-Jupyter: A case study on SUMMA. *Proceedings of the Practice and Experience in Advanced Research Computing on Rise of the Machines (learning). *

[CR28] Mead, M., Popoola, O., Stewart, G., Landshoff, P., Calleja, M., Hayes, M., Baldovi, J., Mcleod, M., Hodgson, T., & Dicks, J. (2013). The use of electrochemical sensors for monitoring urban air quality in low-cost, high-density networks. *Atmospheric Environment,**70*, 186–203.

[CR29] Meehl, G. A., & Tebaldi, C. (2004). More intense, more frequent, and longer lasting heat waves in the 21st century. *Science,**305*, 994–997. 15310900 10.1126/science.1098704

[CR30] Menze, B. H., Kelm, B. M., Masuch, R., et al. (2009). A comparison of random forest and its Gini importance with standard chemometric methods for the feature selection and classification of spectral data. *BMC Bioinformatics,**10*, 213. 10.1186/1471-2105-10-213 19591666 10.1186/1471-2105-10-213 PMC2724423

[CR31] Mohajerani, A., Bakaric, J., & Jeffrey-Bailey, T. (2017). The urban heat island effect, its causes, and mitigation, with reference to the thermal properties of asphalt concrete. *Journal of Environmental Management,**197*(2017), 522–538. 28412623 10.1016/j.jenvman.2017.03.095

[CR32] Moore, N.Y. (2016). The south side: a portrait of Chicago and American segregation. New York City, NY: St. Martin’s Press.

[CR33] Nellore, K., & Hancke, G. P. (2016). A survey on urban traffic management system using wireless sensor networks. *Sensors,**16*, 157. 26828489 10.3390/s16020157 PMC4801535

[CR34] Oh, J. W., Ngarambe, J., Duhirwe, P. N., et al. (2020). Using deep-learning to forecast the magnitude and characteristics of urban heat island in Seoul Korea. *Science and Reports,**10*, 3559. 10.1038/s41598-020-60632-z 10.1038/s41598-020-60632-z PMC7044198 32103119

[CR35] Radhika, Y., & Shashi, M. (2009). Atmospheric temperature prediction using support vector machines. *International Journal of Computer Theory and Engineering,**1*(1), 1793–8201.

[CR36] Rathore, M. M., Ahmad, A., Paul, A., & Rho, S. (2016). Urban planning and building smart cities based on the internet of things using big data analytics. *Computer Networks,**101*, 63–80.

[CR37] Rousseeuw, P. J., & Hubert, M. (2011). Robust statistics for outlier detection. *Wires Data Mining Knowl Discov,**1*, 73–79. 10.1002/widm.2

[CR38] Seto, K., Güneralpa, B., & Hutyra, L. (2012). Global forecasts of urban expansion to 2030 and direct impacts on biodiversity and carbon pools. *PNAS,**109*(40), 16083–16088. 22988086 10.1073/pnas.1211658109 PMC3479537

[CR39] Sharma, A., et al. (2016). Green and cool roofs to mitigate urban heat island effects in the Chicago metropolitan area: Evaluation with a regional climate model. *Environmental Research Letters,**11*, 064004.

[CR40] Shen, H., Huang, L., Zhang, L., Wu, P., & Zeng, C. (2016). Long-term and fine-scale satellite monitoring of the urban heat island effect by the fusion of multi-temporal and multi-sensor remote sensed data: A 26-year case study of the city of Wuhan in China. *Remote Sensing of Environment,**172*, 109–125. 10.1016/j.rse.2015.11.005 ISSN 0034-4257.

[CR41] Shi, Y., Katzschner, L., & Ng, E. (2018). Modelling the fine-scale spatiotemporal pattern of urban heat island effect using land use regression approach in a megacity, Science of The Total Environment, Volume 618, 2018. *ISSN,**891–904*, 0048–9697. 10.1016/j.scitotenv.2017.08.252 ISSN 0048-9697. 10.1016/j.scitotenv.2017.08.252 29096959

[CR42] Somers, K., et al. (2013). Streams in the urban heat island: spatial and temporal variability in temperature. *Freshwater Science,**32*(1), 309–326.

[CR43] Stempihar, J. J., Pourshams-Manzouri, T., Kaloush, K. E., & Rodezno, M. C. (2012). Porous asphalt pavement temperature effects for urban heat island analysis. *Transportation Research Record,**2293*(1), 123–130. 10.3141/2293-15

[CR44] Szymanowski, M., & Kryza, M. (2009). GIS-based techniques for urban heat island spatialization. *Clim Res,**38*, 171–187. 10.3354/cr00780

[CR45] Tan, J. (2010). The urban heat island and its impact on heat waves and human health in Shanghai. *International Journal of Biometeorology,**54*, 75–84. 19727842 10.1007/s00484-009-0256-x

[CR46] Thakur, G., Bhaduri, B., Piburn, J., Sims, K., Stewart, R., & Urban, M. (2015). PlanetSense: a real-time streaming and spatio-temporal analytics platform for gathering geo-spatial intelligence from open source data. *SIGSPATIAL’15: Proceedings of the 23rd SIGSPATIAL International Conference on Advances in Geographic Information Systems* (pp. 1–4)

[CR47] USGS. (2020). *Landsat 8–9 Operational Land Imager (OLI) - Thermal Infrared Sensor (TIRS) Collection 2 Level 2 (L2) Data Format Control Book (DFCB)*. https://d9-wret.s3.us-west-2.amazonaws.com/assets/palladium/production/s3fs-public/atoms/files/LSDS-1328_Landsat8-9-OLI-TIRS-C2-L2-DFCB-v6.pdf.

[CR48] USGS. (n.d.a). *Landsat Level-1 Processing Details*. Retrieved from: https://www.usgs.gov/landsat-missions/landsat-level-1-processing-details

[CR49] USGS. (n.d.b). *Landsat Collection 2 Level-2 Science Products*. Retrieved from: https://www.usgs.gov/landsat-missions/landsat-collection-2-level-2-science-products

[CR50] Wang, S. (2010). A cyberGIS framework for the synthesis of cyberinfrastructure, GIS, and spatial analysis. *Annals of the Association of American Geographers,**100*(3), 535–557.

[CR51] Wang, S. (2016). cyberGIS and spatial data science. *GeoJournal*, *81*(6) , 965–968. 10.1007/s10708-016-9740-0.

[CR52] Wang, S., & Goodchild, M. F. (2019). cyberGIS for Geospatial Innovation and Discovery. *Springer, Dordrecht, Netherlands,*. 10.1007/978-94-024-1531-5

[CR53] Wang, S., Lyu, F., Wang, S., Catlet, C. E., Padmanabhan, A., & Soltani, K. (2021). Integrating cyberGIS and Urban Sensing for Reproducible Streaming Analytics. In W. Shi, M. F. Goodchild, M. Batty, M. P. Kwan, & A. Zhang (Eds.), *Urban Informatics The Urban Book Series. *Singapore: Springer. 10.1007/978-981-15-8983-6_36

[CR54] Wang, S., Zhong, Y., & Wang, E. (2019). An integrated GIS platform architecture for spatiotemporal big data. *Future Generation Computer Systems,**94*, 160–172.

[CR55] Wilson, S. (2021). *miceforest: Fast Imputation with Random Forests in Python*. https://github.com/AnotherSamWilson/miceforest.

[CR56] Wong, M. S., Zhu, R., Kwok, Y. T., Kwan, M. P., Santi, P., Lee, K. H., Heo, J., Li, H., & Ratti, C. (2021). Association between NO2 concentrations and spatial configuration: A study of the impacts of COVID-19 lockdowns in 54 US cities. *Environmental Research Letters,**16*, 054064.

[CR57] Xie, Z. Q., Du, Y., Zeng, Y., Yan, M. L., & Zhu, C. Y. (2010). Accelerated human activities affecting the spatial pattern of temperature in the Yangtze River Delta. *Quaternary International,**226*, 112–121. 10.1016/j.quaint.2010.04.027

[CR58] Yang, J., Wang, Z., & Kaloush, K. (2015). Environmental impacts of reflective materials: Is high albedo a ‘silver bullet’ for mitigating urban heat island? *Renewable and Sustainable Energy Reviews,**47*, 830–843. 10.1016/j.rser.2015.03.092 ISSN 1364-0321.

[CR59] Yin, D., Liu, Y., Hu, H., et al. (2019). cyberGIS-Jupyter for reproducible and scalable geospatial analytics. *Concurrency Computat Pract Exper*, *31*, e5040. 10.1002/cpe.5040.

[CR60] Zhao, L., Lee, X., Smith, R., et al. (2014). Strong contributions of local background climate to urban heat islands. *Nature,**511*, 216–219. 10.1038/nature13462 25008529 10.1038/nature13462

[CR61] Zhou, W., Huang, G., & Cadenasso, M. L. (2011). Does spatial configuration matter? Understanding the effects of land cover pattern on land surface temperature in urban landscapes. *Landscape and Urban Planning,**102*, 54–63.

